# Influence of Resveratrol on the Immune Response

**DOI:** 10.3390/nu11050946

**Published:** 2019-04-26

**Authors:** Lucia Malaguarnera

**Affiliations:** Department of Biomedical and Biotechnological Sciences, University of Catania, Via Santa Sofia 97, 95124 Catania, Italy; lucmal@unict.it

**Keywords:** resveratrol, immune response, macrophages, T lymphocytes, natural killer, B lymphocytes

## Abstract

Resveratrol is the most well-known polyphenolic stilbenoid, present in grapes, mulberries, peanuts, rhubarb, and in several other plants. Resveratrol can play a beneficial role in the prevention and in the progression of chronic diseases related to inflammation such as diabetes, obesity, cardiovascular diseases, neurodegeneration, and cancers among other conditions. Moreover, resveratrol regulates immunity by interfering with immune cell regulation, proinflammatory cytokines’ synthesis, and gene expression. At the molecular level, it targets sirtuin, adenosine monophosphate kinase, nuclear factor-κB, inflammatory cytokines, anti-oxidant enzymes along with cellular processes such as gluconeogenesis, lipid metabolism, mitochondrial biogenesis, angiogenesis, and apoptosis. Resveratrol can suppress the toll-like receptor (TLR) and pro-inflammatory genes’ expression. The antioxidant activity of resveratrol and the ability to inhibit enzymes involved in the production of eicosanoids contribute to its anti-inflammation properties. The effects of this biologically active compound on the immune system are associated with widespread health benefits for different autoimmune and chronic inflammatory diseases. This review offers a systematic understanding of how resveratrol targets multiple inflammatory components and exerts immune-regulatory effects on immune cells.

## 1. Introduction

**Figure 1:**
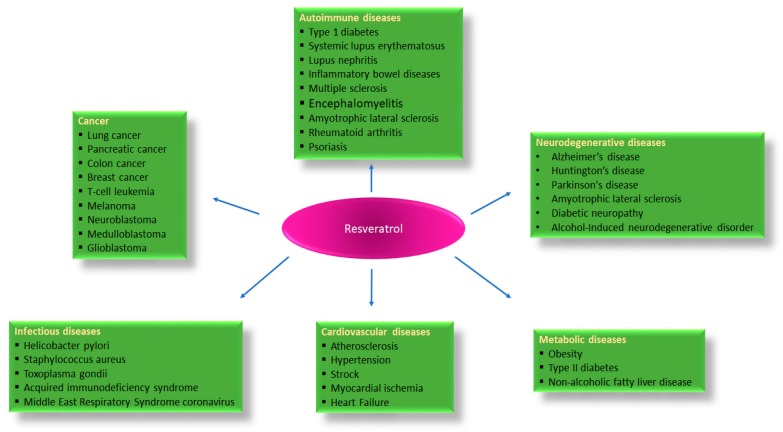
Activity of resveratrol against different human diseases based on experimental studies.

Resveratrol (trans-3,4,5-trihydroxystilbene) is a natural polyphenol found in red wine [1], rhubarb [2], and fruits such as blueberries [3], many red grape varieties [4], and peanuts [5] to name a few, that plays an important role in a large variety of biological activities [6,7]. Resveratrol can exhibit antioxidative, anti-inflammatory, anticancer, antimicrobial, anti-neurodegenerative, and estrogenic properties [8,9]. The immunomodulatory role of resveratrol was proposed 18 years ago, with an investigation that demonstrated how it inhibits the proliferation of spleen cells induced by concanavalin A (ConA), interleukin-2 (IL-2), or alloantigens, and more efficiently prevents the production of IL-2 and interferon-gamma (IFNγ) by lymphocytes and the production of tumor necrosis factor alpha (TNF-α) or IL-12 by macrophages [10]. By interacting with several molecular targets, resveratrol regulates innate and adaptive immunity [11]. Nevertheless, sometimes its properties seem to be contrasting. It has been reported that resveratrol modulates immune function in a dose dependent manner, at low doses resveratrol stimulates the immune system, whereas at high doses it induces immunosuppression [12]. Its effect as an immunomodulator has been demonstrated in various animal models and in different cell lines. In rodents, resveratrol reduces inflammatory responses in peritonitis, reverses immunosenescence in elder rats, and improves immunologic activity against cancer cells [13,14]. Regarding the immune system, it has been found that resveratrol participates in the activation of macrophage, T cell and natural killer (NK), and is involved in CD4^+^CD25^+^ regulatory T cell suppressive functions [11,15]. Its effects are the result of its ability to remove reactive oxygen species (ROS) [16], to inhibit cyclooxygenase (COX) [17,18], and to activate many anti-inflammatory pathways, including among others Sirtuin-1 (Sirt1) [19]. Sirt1 disrupts the TLR4/NF-κB/STAT signal which in turn decreases cytokines production from inactivated immune cells [20], or macrophage/mast cell-derived pro-inflammatory factors, such as platelet-activating factor (PAF), TNF-α, and histamine [21]. For its benefits to human health (Figure 1) and for showing promising properties in immunologic disorders, it is increasingly proposed as a dietary supplement for human consumption [22]. However, the pharmacokinetic analysis reveals that resveratrol undergoes rapid metabolism in the body. Its bioavailability after oral administration is very low, despite absorption reaching 70%, this impacts the physiological significance of the high concentrations used in vitro studies [23].

In the present review, we aim to outline the molecular mechanisms of action, the role in the immunological function, and the therapeutic use of resveratrol in many diseases characterized by inflammation.

## 2. Resveratrol Pathways in Immune Function

**Figure 2:**
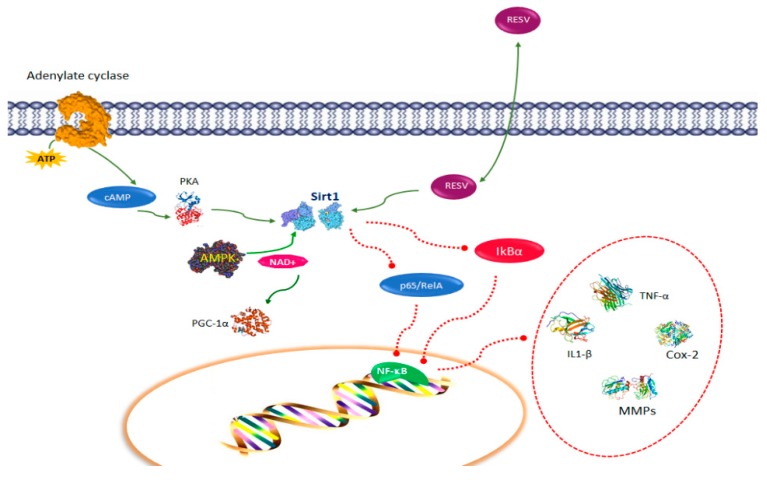
Resveratrol pathways in immune function: resveratrol activates Sirtuin-1 (Sirt1) inhibiting RelA acetylation and promotes inhibitor protein-κBα (IkBα) degradation, which decreases nuclear factor kappa B (NF-κB)-induced expression of tumor necrosis-alpha (TNF-α), interrleukin (IL)-1β, IL(-6), metalloproteases (MMPs), and cyclooxygenase Cox-2. Cyclic adenosine monophosphate (cAMP) levels trigger protein kinase A (PKA), which activates Sirt1. AMP-activated protein kinase (AMPK) controls the activity of Sirt1 by regulating the cellular levels of nicotinamide adenine dinucleotide (NAD^+^). In the downstream activation of AMPK, an increase of NAD^+^ levels induces Sirt1 activation, which promotes deacetylation and activation of peroxisome proliferator-activated receptor gamma coactivator 1-alpha (PGC-1α).

A key function of resveratrol is to inhibit the production of inflammatory factors through the activation of Sirt1 [24]. Sirt1 is an important deacetylase involved in numerous molecular events, including metabolism [25], cancer [26], embryonic development [27], and immune tolerance [28,29]. Sirt1 maintains periphery T cell tolerance. The ablation of Sirt1 leads to the enhancement of T cell activation and the occurrence of spontaneous autoimmune disease [30]. Structural studies indicate that resveratrol binding to Sirt1 modulates the Sirt1 structure and enhances binding activity to its substrates [31]. Due to its aptitude to activate Sirt1 and suppress inflammation, resveratrol is able to alleviate inflammatory symptoms in several experimental autoimmune disease models, such as colitis, type I diabetes, encephalomyelitis, and rheumatoid arthritis [32,33] (Figure 1). One of the principal substrates of Sirt1 is p65/RelA [34], a NF-κB member, which is the major regulator of leukocyte activation and inflammatory cytokines signaling [35]. The activation of Sirt1 by resveratrol generates the inhibition of RelA acetylation, which in turn decreases NF-κB-induced expression of inflammatory factors such as TNF-α, IL-1β, IL-6, metalloproteases (MMP)-1 and MMP3, and Cox-2 [36] (Figure 2). Resveratrol-treated cells are less responsive to TNF-α-induced NF-κB signaling and apoptosis initiation, acting as a double block on the NF-κB signaling pathway [37]. Moreover, resveratrol inhibits p300 expression and promotes inhibitor protein-κBα (IkBα) degradation, nevertheless, it is unknown whether this process occurs through Sirt1 activation [38]. The targets of resveratrol include AMP-activated protein kinase (AMPK), an essential energy sensor in cells, which controls the activity of Sirt1 by regulating the cellular levels of available nicotinamide adenine dinucleotide (NAD^+^) [39] (Figure 2). Cyclic adenosine monophosphate (cAMP) levels trigger protein kinase A (PKA), which in turn phosphorylates and activates Sirt1 [40]. The evidence that the function of resveratrol is mediated, in part by Sirt1, is confirmed by the observation that the anti-inflammatory properties of resveratrol are abolished by the genetic deletion of Sirt1, or by the addition of Sirt1 inhibitors such as Sirtinol [39,40,41]. In the downstream activation of AMPK, an increase of nicotinamide adenine dinucleotide (NAD^+)^ levels induces Sirt1 activation, which promotes beneficial metabolic changes primarily through deacetylation and activation of peroxisome proliferator-activated receptor gamma coactivator 1-alpha (PGC-1α) (Figure 2).

## 3. Resveratrol and Macrophages

**Figure 3:**
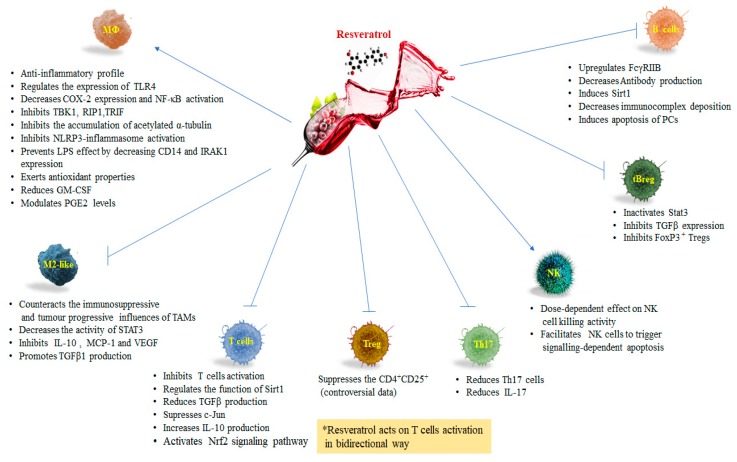
Effects of resveratrol on immune cells: Breg, regulatory B cell; COX2, cyclooxygenase; FOXP3, forkhead box P-3; GM-CSF, granulocyte–macrophage colony-stimulating factor; IL-10, interleukin-10; IL-17, interleukin 17; IRAK, interleukin-1 receptor-associated kinase; LPS, lipopolysaccharide; MΦ, macrophage; MCP1, monocyte chemoattractant protein-1; NF-κB, nuclear factor-Kappa B; NLRP3, nod-like receptor family, pyrin domain containing 3; Nrf2, nuclear factor erythroid 2-related factor 2; RIP, receptor-interacting protein; PCs, plasma cells; PGE2, prostaglandin E2; Sirt1, silent mating type information regulation 2 homolog; STAT3, signal transducer and activator of transcription; TAMs, tumor associated macrophages; TBK1, TANK-binding kinase1; TGF-β1, transforming growth factor-β1; Treg, regulatory T cell; Th17, T helper 17; TRIF, toll-interleukin-1 receptor domain-containing adaptor inducing interferon; TLR-2, toll-like receptor-2; VEGF, vascular endothelial growth factor.

Resveratrol exhibits an anti-inflammatory profile in macrophages [35]. Macrophages differentiate from blood monocytes and participate in both innate and adaptive immunity. These cells constitute a heterogeneous pool of cells with a wide range of biological activities depending on their physical location and on external signals received from the tissue microenvironment [19]. Many of these activities appear to be divergent in nature: pro- versus anti-inflammatory effects, immunogenic versus tolerogenic activities, and tissue destruction versus tissue repair [42]. With a wide range of pattern recognition receptors (PRRs), these immune cells can specifically identify conserved pathogen-associated molecular patterns (PAMPs), which are exclusively present on microbes such as viruses, bacteria, parasites, and fungi [43]. The main members of PRR families are transmembrane TLRs, C-type lectin receptors (CLRs), cytoplasmic nucleotide oligomerization domain (NOD)-like receptors (NLRs), and RNA helicase retinoic acid inducible gene I (RIG-I)-like receptors (RLRs). Thus, the activated intracellular signaling induces the expression of genes involved in inflammatory and/or immune response and the recruitment of phagocytic cells to the site of infection. Because of their abilities to recognize pathogens and activate bactericidal activities, macrophages are always operating at the site of immune defense [44]. They produce anti-inflammatory cytokines, such as interleukin-10 (IL10) and transforming growth factor-beta (TGF β), and inhibit the inflammatory pathways mediated by TLRs [45]. TLRs initiate signaling in innate and adaptive immune pathways. This highly conserved family of transmembrane proteins comprises an extracellular domain that recognizes exogenous and endogenous danger molecules and an ectodomain that activates downstream pathways. Several lines of evidence suggest that continuous activation or dysregulation of TLRs signaling may contribute to the occurrence of chronic pathological conditions [46]. Resveratrol regulates the expression of TLR4 [47]. Hence, resveratrol can be employed for TLR-mediated inflammatory responses and chronic diseases linked to TLR activation including obesity, type 2 diabetes mellitus (T2DM), fatty liver disease, Crohn’s disease, rheumatoid arthritis, cardiovascular and neurodegenerative disorders (Figure 1) [48]. The molecular regulation of inflammatory response is substantially modulated by transcription factors. It has been demonstrated that resveratrol decreases NF-κB activation and COX-2 expression in lipopolysaccharide (LPS)-induced RAW264.7 (Figure 3). Moreover, it inhibits TANK-binding kinase1 (TBK1) and receptor-interacting protein 1 (RIP1) in a toll-interleukin-1 receptor domain-containing adaptor inducing an interferon (TRIF) complex in myeloid differentiation factor 88 (MyD88)-independent signaling pathways (Figure 3) [49]. Additional studies showed that resveratrol exerts anti-inflammatory effects by attenuating TLR4-TRAF6, mitogen-activated protein kinase (MAPK), and AKT pathways in LPS-induced macrophages [50]. Another signaling pathway that has been linked to inflammation is the endoplasmic reticulum (ER) response [51]. ER stress leads to the activation of inositol-requiring enzyme 1 (IRE1), which splices X-box binding protein 1 (Xbp-1) into its functional message, and ultimately leads to suppressed global translation and increased chaperone activity. If the cells fail to reduce the ER load, they will undergo apoptosis [52]. It has been suggested that the IRE1α-Xbp-1 pathway is critical for TLR-induced inflammatory cytokines production by macrophages [53]. Xbp-1 is regulated by post-translational acetylation and deacetylation mediated by the acetyltransferase p300 and deacetylase Sirt1, respectively [54]. More recently it was shown that resveratrol prevents the increase of acetylated α-tubulin caused by mitochondrial damage in macrophages stimulated with inducers of the nod-like receptor family, pyrin domain containing 3 (NLRP3) inflammasome (Figure 3). As result, since resveratrol influences the generation of an optimal site for the assembly of NLRP3 and ASC and, in turn, inhibits NLRP3-inflammasome activation, it could be an effective medication for the treatment of NLRP3-related inflammatory diseases [55]. Resveratrol not only influences the transcription of NF-κB elements but also the transcription of STAT1 and cyclic adenosine monophosphate (cAMP)-responsive element binding protein 1 (CREB1) [56]. TNF-α-induced activation of the NF-κB elements is modulated by resveratrol. Interestingly, a study reported that LPS dose-dependently increased extracellular malondialdehyde (MDA) and nitric oxide without affecting their intracellular level, whereas resveratrol abolished all these deleterious effects. LPS-activation of monocytes and macrophages induces the NF-κB dependent transcription of chemokines such as CXCL8/IL-8, CXCL10/IP-10, CCL2/MCP1, and CCL5/RANTES [45]. LPS increased CD14 expression, interleukin-1 receptor-associated kinase (IRAK1), and a phosphorylated form of p38 MAPK protein. Resveratrol prevented LPS effect by decreasing CD14 and IRAK1 (Figure 3) expression but, surprisingly, increased the p38 MAPK protein phosphorylation [57]. Further investigation showed that resveratrol decreased LPS-induced pro-oxidant effect in the AR42J cell line via a Myd88-dependent signaling pathway. This data indicated that resveratrol exerted antioxidant properties by a Myd88-dependent way not involving IRAK1 or by a TRIF dependent pathway [57] (Figure 3). Sirt1 has a direct regulatory role in macrophages functions during inflammation. The production of pro-inflammatory cytokines TNF-α, IL-6, and IL-1β by macrophages from the myeloid-specific Sirt1 knockout mice is dramatically increased in response to infection and inflammation. In addition to pro-inflammatory cytokines, Sirt1 is involved in the expression of cell surface molecules such as intercellular cell adhesion molecule1 (ICAM-1) to facilitate macrophage trafficking during the inflammatory response (Figure 3) [58]. Hyperacetylation of NF-κB transcription factor RelA/p65 has been detected in macrophages from myeloid-specific Sirt1 knockout mice, indicating that the anti-inflammatory activity of Sirt1 in macrophages occurs, at least partially, through NF-κB suppression [59].

Remarkably, resveratrol also stronglyreduces the production of granulocyte-macrophage colony-stimulating factor (GM-CSF) [60] (Figure 3), a pro-inflammatory cytokine that acts at the interface between innate and adaptive immunity essential for survival/differentiation/activation of pro-inflammatory macrophages [61] and a key marker of atheroma formation [62]. Several evidences indicate that resveratrol modifies cell morphology, gene expression, ligand-receptor interactions, signaling pathways, and foam-cell formation [63,64,65]. Additionally, resveratrol modulates the immune response by influencing cellular prostaglandin E2 (PGE_2_) levels (Figure 3). PGE_2_ plays an important role in the regulation of the immune response [66]. Resveratrol up-regulates COX-2 in various inflammatory diseases [66,67]. The cell-specific effect on interleukin production is another important function of resveratrol. In fact, resveratrol was found to enhance the expression of IL-1β and IL-6 in the peripheral blood lymphocytes (PBLs), but it had opposite effects in macrophages [60]. The enhanced production of IL-1β and IL-6 characterizes a pro-inflammatory status contributing to T helper lymphocytes differentiation and function [68], but it is also involved in tissue regeneration [69]. The immune cells exposed to resveratrol in the vascular compartment expressing significant levels of IL-1β or IL-6 are triggered for the adaptive immune response. Nevertheless, resveratrol affects the immune cells only for a limited time because of its short half-life in the blood [70]. These findings suggest that resveratrol facilitates the systemic response to injuries [10,45] and restrains the low-grade inflammatory status related to chronic diseases in tissues.

### Resveratrol and Tumor Associated Macrophages (TAMs)

Clinical and experimental evidence reports that high infiltration of macrophages in most human cancers is associated with tumor malignancy, poor prognosis, and tumor relapse. Macrophages show plasticity in their activation profile under different cytokines stimulation. They are capable of both inhibiting and promoting the growth and spread of cancers, depending on their activation state. Macrophages can be classically activated (M1), in the presence of IFN-γ and LPS, while in the presence of IL-4 and IL-13, or indirectly through Th2 cells induction toward alternatively activated macrophages (M2). Macrophage polarization deeply alters the immune properties of these cells [44]. The M1 polarized macrophages manifest high levels of proinflammatory cytokines and promote Th1 responses, which contributes to tumoricidal activity and antitumor immunity [42]. The polarization of M1 macrophages is mainly regulated by distinct transcriptional networks consisting of an interferon regulatory factor (IRF-1/5), STAT-1/4, and NK-κB [71,72]. Alternatively, M2-like polarization of macrophages, which produce secretory factors to promote tissue remodeling, immune tolerance, and angiogenesis may be linked with tumor progression. M2 polarization is induced by Th2 cytokines, like IL-13 and IL-4 [72], and is regulated by transcription factors such as IRF-4, STAT-3/6, PPAR-γ, and Krüppel-like factor 4 (KLF-4) [73,74,75]. An accumulating line of evidence indicates that macrophages ruling to execute tumor-promoting or tumor-suppressing activities depend on their sub-phenotype, which is dynamically switched [76,77]. TAMs in malignant tumors resemble alternatively activated macrophages (M2-like). They enhance tumor-associated angiogenesis, promote tumor migration and invasion, and lack in anti-tumor immune responses [78]. A high density of TAMs, particularly the M2 subset, matches to worse overall survival (OS) in patients with lung cancer, gastric cancer, or breast cancer [79,80,81]. TAMs infiltrated in primary tumors or metastatic sites have a critical role in directing tumor cells from the primary site to distant tissues in different murine models [82,83]. TAMs in the peripheral blood may mediate circulating tumor cells migration and aid their achieving into distant metastatic sites [84]. In an experimental in vitro model investigating macrophage morphology and functions in relation to the tumor microenvironment, it was observed that treatment with a synthetic resveratrol analogue HS-1793 significantly increased IFN-γ, which reprogrammed the M2 phenotype (Table 1). Therefore, it was proved that HS-1793 powerfully counteracted the immunosuppressive and tumor progressive influences of TAMs [85]. STATs are cytoplasmic transcription factors that act as intracellular effectors of cytokine and growth factor signaling pathways. STAT3, a member of STAT family, plays a key role in promoting proliferation, differentiation, anti-apoptosis, or cell cycle progression. Constitutive activation of STAT3 is involved in a variety of tumor cells [86]. As mentioned before, activation of STAT3 in the M2 subset leads to tumor-induced immunosuppression and constitutively activates STAT3 inhibiting the expression of mediators required for immune activation against tumor cells. In several murine models of carcinogenesis, tumor progression is frequently associated with a phenotypic switch from M1 to M2 in TAMs [76]. The inhibition of STATs signaling pathways can suppress tumor growth and metastasis by inhibiting M2-like polarization of macrophages, further suggesting that TAMs are a possible target in cancer therapy [87]. To date, many studies have been carried out about the roles of STAT3 in cancer and therapeutic applications. In lung cancer cells resveratrol treatment decreases the activity of STAT3 and inhibits lung cancer progression by suppressing the pro-tumor activation of TAMs [88] (Figure 3). In addition, in a mouse lung cancer xenograft model, resveratrol significantly inhibits the tumor growth, decreasing cell proliferation and expression of p-STAT3 in tumor tissues [88]. Other studies demonstrated that both antitumor and antimetastatic effects of resveratrol were partly due to anti-lymphangiogenesis through the regulation of M2 macrophage activation and differentiation [89]. In fact, resveratrol inhibited the production of IL-10 of monocyte chemoattractant protein-1 (MCP-1) in M2 macrophages, whereas it promoted TGF β1 production. Nevertheless, resveratrol inhibited the phosphorylation of STAT3 without affecting its expression in the differentiation process of M2 macrophages. Furthermore, a resveratrol-treated condition medium of M2 macrophages inhibited vascular endothelial growth factor C (VEGFC)-induced human lymphatic endothelial cells (HLECs) migration, invasion, and lymphangiogenesis (Figure 3). In vivo resveratrol inhibited tumor growth and metastasis to the lung, and reduced the area of lymphatic endothelial cells in tumors [89].

## 4. Resveratrol and T Lymphocytes

The improvement of effective adaptive immunity is much more persistent and reliant on the responses of T and B lymphocytes cooperating with antigen presenting cells (APC) in peripheral lymphoid tissue over the course of days and weeks. However, once the adaptive immune responses occur, Th1 and Th17, subsets of effector T helper cells, migrate from lymphoid tissue into circulation, infiltrate infected sites, and produce their own cytokines enriching macrophages and neutrophils activity, respectively. Both innate and adaptive immunity possess the ability to control inflammation and develop self and non-self-discrimination. During development, immature T cell populations acquire the ability to express antigen-specific receptors that distinguish self or non-self-macromolecules [90]. In the thymus, developing T lymphocytes with T cell receptors (TCRs) are capable of high affinity recognition of self-peptides in the context of self, and major histocompatibility complex (MHC) proteins undergo apoptosis in a negative selection [91]. As a safeguard against self-reactive T cells entry into periphery lymphoid tissue, regulatory T cells (Tregs) are produced naturally (nTregs) during central development of T cells in the thymus [92] and are induced peripherally (iTregs) during the progression of immune responses [93]. Failures of tolerance within the adaptive immune system are uncommon. But, when central and/or induced peripheral tolerance fail, autoimmune diseases can arise. Abnormal T cell activation is involved in many autoimmune diseases, such as insulin-dependent diabetes, rheumatoid arthritis, systemic lupus erythematosus, and multiple sclerosis [94]. Given that resveratrol can inhibit T cell activation and reduce cytokine production, it is conceivable that it can prevent autoimmune disease progression. It has been reported that resveratrol-treated mice displayed significantly reduced disease incidence and footpad thickness. Histological analysis demonstrated that infiltrated cells in the joint were clearly reduced in the resveratrol-treated mice compared with the control mice. This observation indicated that resveratrol can prevent the development of collagen-induced arthritis [33]. The Th17 cells are CD4+ T subsets, their development depends on signals mediated by IL-6, TGF-β, IL-21, and IL-23, and by induction of the lineage-specifying transcription factor, retinoic acid-related orphan nuclear receptor (ROR*γ*T). Unlike Th1 and Th2 cells, which after differentiation are secretory cells, Th17 cells maintain their stem cell–like properties, which allows them to persist for a long time while retaining the aptitude to produce functionally divergent progeny when reactivated by antigen [95]. The Th17 cells are key initiators of proinflammatory responses, by recruiting neutrophils and macrophages to injured tissues, and via their production of IL-17 play an important role in host defense against infection to extracellular pathogens. An additional cytokine produced by Th17 is IL23, which controls survival and maintenance of the Th17 phenotype and is responsible for the crosstalk between innate and adaptive immunity [96]. Moreover, Th17 cells produce IL-22 which, similar to IL-17, is beneficial to the host in many infectious and inflammatory disorders. Nevertheless, synergistically with IL-17, it can play an important role in disease due to its proinflammatory properties [97]. Th17 cells are powerful inducers of chronic inflammatory responses. Th17 cells play very important roles in autoimmune disease [96]. Resveratrol could modulate murine collagen-induced arthritis by inhibiting Th17 and B-cells function [33]. The arthritis-protective effects of resveratrol are also associated with the reduced numbers of Th17 cells and the production of IL-17 in the draining lymph node (Figure 3) [33]. Resveratrol protection against experimental autoimmune encephalomyelitis (EAE) is not associated with declines in IL-17^+^ T cells but is associated with rises in IL-17 ^+^/IL-10^+^ T cells and CD4-IFN-γ^+^ and with repressed macrophage IL-6 and IL-12/23 p40 expression [98]. Interestingly, the function of resveratrol on Treg cells seems to benefit from T cell activation. CD4^+^CD25^+^Foxp3^+^ cells were found significantly reduced in the total splenocytes as in tumor tissues from HS-1793-administered mice, and the production of TGF-β inducing Treg showed a similar pattern [99]. The administration of resveratrol suppresses the CD4^+^CD25^+^ cell population among CD4^+^ cells, down-regulates the secretion of TGF-β, and enhances IFNγ expression in CD8+ T cells both ex vivo and in vivo, leading to immune stimulation [16]. Other findings established that resveratrol decreases the expressions of CD28 and CD80 and increases the production of IL-10, but does not influence the percentage of CD4^+^CD25^+^ Treg cells [11] (Figure 3). Therefore, the studies reporting the effects of resveratrol on T cells and its specific molecular mechanisms are in some cases controversial. Sirt1 is involved in periphery T cell tolerance [23], ablation of Sirt1 in T cells could induce hyper-activation of T cells and lead to spontaneous autoimmune disease (Figure 3) [30]. It has been reported that resveratrol inhibits T cell activation and production of antigen-specific antibody in vivo. The inhibition of T cell activation by resveratrol is mediated by Sirt1 as demonstrated by the observation that the inhibitory effect of resveratrol on T cell activation disappeared in Sirt1 knockdown T cells. Moreover, Sirt1 expression was up-regulated in activated T cells and was higher in resveratrol-treated T cells than in naïve T cells. Other data demonstrated that resveratrol maintains T-cell tolerance in mice by regulating the function of Sirt1 which inhibits activation of self-reactive T cells that escape negative selection in the thymus [100]. The mechanism by which resveratrol modulates T cell activation has been, in part clarified. Resveratrol increased Sirt1 acetylase activity on c-Jun, but not on the nuclear factor of activated T cells (NFAT) and NFκb in T cells [101]. However, resveratrol cannot decrease the acetylation of c-Jun in Sirt1^-/-^ T cells, strongly suggesting that acetylation change of c-Jun is totally dependent on Sirt1. Once T cells are activated, c-Jun translocate into the nucleus. Nevertheless, the action of c-Jun is suppressed in resveratrol-treated T cells (Figure 3). Thus, resveratrol can clearly inhibit T cell activation by increasing the expression of Sirt1 and the deacetylase activity of Sirt1 on c-Jun, which in turn blocks the translocation of c-Jun into the nucleus [102]. Additionally, resveratrol represses the protein kinase Cθ in peripheral blood T lymphocytes in a rat liver transplantation model [103].

Obesity has deleterious effects on cell-mediated immunity and increases the risk of infectious diseases. In facts, obesity dysregulates T-cell generation and function impairing the ability to promote a peripheral T-cell-mediated protective immune response and damages wound healing and infection [104,105]. Several studies in murine models have elucidated how resveratrol can reverse the deleterious effects of T-cell function in diet-induced obesity. Interestingly, resveratrol as a supplement for a high-fat diet (HFD) relieves oxidative stress, inhibits inflammatory genes expression, and increases Tregs number via aryl hydrocarbon receptor activation in HFD-induced obese mice [106]. Furthermore, resveratrol decreases the fasting blood glucose and fasting plasma insulin and increased the CD3^+^CD4^+^/CD^3^+CD8^+^ subsets percentages in the obese model of C57BL/6 mice [106] (Figure 1). Interestingly, the reduction in CD3^+^CD4^+^/CD^3^+CD8^+^ ratio is usually associated with malignancies or the attack of the virus such as HIV infection [107] (Figure 1). This reduction also existed in the mouse model of systemic lupus erythematosus [108], suggesting that resveratrol may act in these diseases inducing CD3^+^CD4^+^/CD^3^+CD8^+^. Moreover, resveratrol activates the nuclear factor erythroid 2-related factor 2 (Nrf2) signaling pathway-mediated antioxidant enzyme expression and alleviates the inflammation by protecting against oxidative damage and T-lymphocyte subset-related chronic inflammatory response in the development of HFD-induced obesity [106] (Figure 1). The data suggested that resveratrol supplement-maintained glucose homeostasis by activating the phosphatidylinositol 3’-kinase (PI3K) and SIRT1 signaling pathways. Overall, these evidence indicate that resveratrol can be used in clinic for treating inflammation induced by T cell activation and other T cell-related diseases. Resveratrol acts on T cells activation in a bidirectional way: for autoimmune disease model it exerts an inhibitory function, whereas for tumor model resveratrol reduces the suppressive function of Tregs inhibiting the tumor growth.

## 5. Resveratrol and Natural Killer Cells

NK cells comprise about 15% of all circulating lymphocytes [109] and are able to lyse cancer cells in vitro without prior immune sensitization [110]. Their main importance resides in early host defense against both allogenic and autologous cells after virus infection [111], infection with bacteria or parasites, or against tumor cells [112]. NK cells express various PRRs like TLRs, NLRs, and RLRs. They respond to PAMPs in a suitable milieu in the presence of cytokines like IL-2, IL-12, IL-15, or IL-18. Consequently, activated NK cells release IFN-γ, GM-CSF, TNF-α or cytotoxic granules directed toward a target cell. NKs kill target cells through different mechanisms. Firstly, NK cells form immune synapses. Afterward, they release cytoplasmic granules, organelles containing perforin (Prf1), the saposin-like family member granulysin, and serin-proteases such as granzyme B (GzmB) to cleave several pro-caspases, which trigger apoptosis in the target cell [113]. As well, the expression of members of the tumor necrosis factor (TNF)-family such as FAS ligand (FASL), TNF, and TNF-related apoptosis inducing ligand (TRAIL) induce tumor-cell apoptosis upon formation of immune synapses. Another mechanism of action to kill target cells is the secretion of a number of effector cytokines such as IFN-γ, IL-5, IL-10, IL-13, and GM-CSF after achievement of distinct stages of NK-cell differentiation. NK cells secrete also a variety of chemokines including chemokine C-C motif ligand (CCL) such as CCL2, CCL3, CCL4, CCL5, monocyte-chemoattractant protein (MCP-1), macrophage inflammatory protein (MIP-1α), and (MIP-1β), RANTES, chemokine X-C motif ligand 1 (XCL1, lymphotactin), and IL-8. NKs interacting with other immune cells like dendritic cells in areas of inflammation modulate the innate and adapatative immune response and promote T-cell response against tumors [114]. Their killing capacity against malignant cells depends on stimulation of two main structural classes of NK cell surface receptors such as receptors of the C-type lectin-like family and the killer cell immunoglobulin-like receptors (KIRs), which inhibit and/or activate signaling cascades. Certain human activating receptors like different KIRs or natural cytotoxicity receptors (NCRs) such as NKp30, NKp44, NKp46, and NKp80 activate signal via protein tyrosine kinase-dependent pathways. To antagonize NK cell activation, inhibitory surface receptors like different KIRs in humans are present, which act through protein tyrosine phosphatase-dependent pathways [115]. Resveratrol exerts a direct influence on the ability of killing of NK cells and simultaneously affects other immune cells like CD8^+^- and CD4^+^-T-cells [116]. Resveratrol possesses therapeutic potential in boosting NKs activity against aggressive cell leukemia and lymphomas by inhibiting constitutively active signal transducers and activators of transcription 3 (STAT3) signaling [117]. NK cell killing capacity has been detected in human immortalized myelogenous leukemia K562 cells. NK cell cytotoxic activity was enhanced at low resveratrol concentration whereas at high concentration it was suppressed (Table 1) [116].

Other findings demonstrated inhibition of viability and increased apoptosis of NK cells upon incubation with high resveratrol concentrations, whereas low concentrations induced an upregulation of NKG2D and IFN-γ and increased NK cell killing towards leukemia K562 target cells [118] (Figure 3). These data suggest a concentration-dependent biphasic effect of resveratrol, which is caused by stimulating cell apoptosis via caspase signaling pathways in high concentration ranges. As supported by a significantly reduced rate apoptotic/necrotic cells after pre-treatment with the caspase inhibitor z-VAD-FMK. In addition, this study showed a higher cytotoxic susceptibility of Jurkat cells, a human lymphoblastoid T cells line, towards resveratrol. A similar dose-dependent enhancement of cytotoxic NK cell killing activity was also observed against tumor cell lines derived from solid tumors, such as HepG2 and A549 cells after pre-stimulation of immortalized NK cells (NK-92 cells) with resveratrol at low concentrations [13] (Table 1). Further, has been reported that in NK-92 resveratrol-treatment induces phosphorylation of ERK-1/2 and JNK and a dose-dependent upregulation of perforin expression [119]. An increase NK cell killing activity with a consequent anticancer effect was observed in a study evaluating the anti-infectious properties of resveratrol in a murine acute pneumonia model [120]. The resveratrol-treated group showed an increased alveolar macrophage infiltration, an elevated NK cell activity, a decreased bacterial burden in the lungs and a decreased mortality. Remarkably, isolated spleen NK cells of rats pre-treated with resveratrol displayed an enhanced killing efficacy against YAC-1 target cells. As well, resveratrol treatment makes promyeloblastic leukemia KG-1a cells susceptible to cytokine-induced killer-mediated cytolysis via an increase in cell-surface expression of natural group 2, member D (NKG2D) ligands and receptor DR4, combined with a downregulation of cell-surface expression of DcR1 in KG-1a cells, and an activation of the TNF-related apoptosis-inducing ligand (TRAIL) pathway [121] (Figure 2). Resveratrol upregulates the agonistic receptors DR4 and DR5 in androgen-insensitive human prostate carcinoma cells PC-3 and DU-145 [122], enhancing TRAIL sensitivity and possibly facilitating NK cell-mediated killing. Similar results were obtained in human prostate adenocarcinoma LNCaP cells and on PC-3 prostate cancer cells TRAIL-resistant that after treatment with resveratrol showed an enhancement of DR4 and DR5 surface expression. A dose-dependent activation of caspase-3 for resveratrol treatment alone, and caspase-8 activation for combined treatment with resveratrol and TRAIL was observed as well. Human 1205 LU metastatic melanoma cells show a resveratrol-dependent enhanced sensitivity to TRAIL through downregulation of the antiapoptotic proteins cellular FLICE-like inhibitory protein (cFLIP) and Bcl-xL [123]. Moreover, resveratrol sensitizes to TRAIL-induced apoptotic cell death various other cancer cells types such as pancreatic, breast, colon cancers, T-cell leukemia, melanoma neuroblastoma, medulloblastoma, and glioblastoma [124]. Resveratrol is able to increase CD95L expression on HL60 human leukemia cells and on T47D breast carcinoma cells [125] (Figure 3) facilitating NK cells to trigger signaling-dependent apoptosis. Because of tumor cell-platelet aggregation, circulating tumor cells coated by aggregated platelets could escape the immune response aiding the occurrence of metastasis. Cancer cells can activate platelets and their aggregation, which correlates with their metastatic potential [126]. A connection of platelet aggregation and the susceptibility of cancer cells to NK cell-mediated lysis has been reported [127]. Remarkably, resveratrol inhibits platelet aggregation via reduction of integrin gpIIb/IIIa on the platelet membrane, which acts as a fibrinogen receptor involved in clot formation that generates bridges between platelets. Resveratrol reduces the production of TxA2, which activates platelets and so exacerbates aggregation, through inhibition of COX1-dependent pathways [128].

## 6. Resveratrol and B Lymphocytes

B cells are characterized by their capacity to produce antibodies. As well, they release cytokines and act as secondary APCs. B cells possess distinct subpopulations that accomplish both regulatory and pathogenic functions. Regulatory B cells (Bregs) are a rare B cell subpopulation (less than 10% of total B-cells in circulation) with regulatory/suppressor functions and are important for the peripheral tolerance mechanisms [129]. Their regulatory activity is generally, but not exclusively performed through IL-10 production. Less than 20% of these cells from the different subsets are IL-10 producers after stimulation [130]. Inflammation induces potently Bregs development and differentiation. A combination of different molecules including TLRs, CD40, the B cell receptor, CD80, CD86, and cytokines are required to activate Bregs [129]. Three different types for Breg cells have been characterized on the basis of activation pathways: innate Breg cells requiring signaling via innate receptors, such as TLRs; immature Breg cells requiring CD40 stimulation; antigen-specific Breg cells requiring both B-cell receptor and CD40 signaling. Bregs prevent inflammation by inhibition of Th1 cells activation, maintenance of the Treg cell population and Th17 proliferation and differentiation [131]. Although IL-10 is a key player in Breg inhibition of inflammation, new investigations have shown that some Breg subsets perform their suppressive function through additional factors. It has been reported that cancer metastasis needs the involvement of regulatory immune cells, such as FoxP3^+^CD4^+^ Tregs and TGFβ-expressing tBregs [132]. Tregs and tBregs need to be controlled to efficaciously prevent lung metastasis. Low doses and non-cytotoxic doses of resveratrol prevents progression of B16 melanoma and of 4T1.2 breast cancer cells and abrogates lung metastasis by inactivating tBregs, thereby disabling tBregs ability to convert FoxP3^+^ Tregs, a process that requires TGFβ expression (Figure 2) [133]. Moreover, resveratrol at a low and non-cytotoxic dose inhibits the generation and function of tBregs by inactivating Stat3 (Table 1). This inactivation of Stat3 in tBregs probably causes the inhibition of TGFβ expression, a downstream target of Stat3 [133] (Figure 3).

This study suggested that low doses of resveratrol can be used to induce antitumor effector and to combat cancer escape mediated by tBregs. [133]. Recently it has been demonstrated that resveratrol treatment can ameliorate lupus nephritis in MRL/lpr mice by upregulating FcγRIIB, leading to a selective reduction of B cells in the spleen and bone marrow [134]. Moreover, plasma cells, expressing the highest levels of FcγRIIB were significantly reduced in both spleen and bone marrow in response to resveratrol (Figure 3). Depletion of autoreactive plasma cells caused a decrement of autoantibody production, thereby leading to decreased immune complexes deposition in the kidney [135]. This result is of clinical importance because neither anti-proliferative agents, for example, cyclophosphamide, nor anti-CD20 mAbs, such as rituximab, can efficiently eliminate plasma cells from the bone marrow of systemic lupus erythematosus (SLE) patients [134] (Figure 3). Moreover, it was shown that Sirt1 induced by resveratrol inhibits B cells proliferation and autoantibody production (Figure 3) ameliorating SLE in a mouse model with constitutive and continued activation of Th1 cells [136].

Lupus nephritis is characterized by glomerular and tubulointerstitial inflammation and mesangial cell proliferation, followed by progressive glomerulosclerosis and interstitial fibrosis between tubules. Resveratrol significantly reduced fibrosis in both glomeruli and tubulointerstitial space, and significantly restored glomerular morphology [136]. In addition, the degree of immunocomplexes deposition in the glomerulus was markedly reduced. The inhibitory effects of enhanced FcγRIIB expression on B cells in vivo may allow FcγRIIB to execute a self-regulatory feedback loop to control the number of plasma cells via immunocomplex-dependent apoptosis. This effect is of clinical relevance in that reduced surface FcγRIIB expression on memory B cells and PCs is often observed in SLE patients, leading to a limited capacity to restrain B cells from activation and to induce apoptosis of PCs (Figure 3) [137]. Therefore, the pharmacological upregulation of FcγRIIB expression by resveratrol can produce a significant decrease of PCs and autoantibody production. This data indicated that the depletion of autoreactive PCs in the bone marrow after resveratrol treatment is mainly mediated by the FcγRIIB-dependent apoptotic pathway rather than inhibition of B cell receptor (BC)R-dependent activation [136]. Other studies have corroborated the idea that elimination of PCs, mainly long-lived PCs in the bone marrow, is crucial in the treatment for SLE patients [137] (Figure 1). Clinically, the upregulation of FcγRIIB in B cells could be of particular benefit for improving the outcome of SLE patients who manifest downregulation of surface FcγRIIB on their memory B cells and PCs [134]. In addition, it was demonstrated that NF-κB is a critical regulator of resveratrol in the upregulation of FcγRIIB expression [134]. Because neither T cells nor NK cells express FcγRIIB, the selective modulation on humoral immunity via FcγRIIB, emphasize an exclusive therapeutic strategy for SLE, without affecting other immune functions and avoiding the side effects of systemic immunosuppression induced by current treatments [137].

## 7. Conclusions

There is an abundance of experimental studies highlighting the regulatory mechanisms and the immunomodulatory role of resveratrol both in vivo and in vitro. These data reveal the promising role of resveratrol in the prevention and therapy of a wide variety of chronic diseases including cardiovascular, inflammatory, metabolic, neurological and skin diseases, and various infectious diseases (Figure 1). There are also increasing lines of evidence suggesting it has a potent chemosensitizing effect in various cancers. These studies show that resveratrol modulates many cellular and molecular mediators of the inflammatory response. Nevertheless, a few studies have reported that resveratrol can function as an antagonistic as well. Its effects are context-dependent (i.e., resveratrol might influence chemokines and cytokines in opposite ways in different tissues). Although, preclinical studies produced exciting results, nowadays many questions remain unanswered about the usage of resveratrol in the clinical setting just because the clinical evidence indicating that resveratrol is an effective therapeutic in humans are still lacking. Moreover, some official systematic clinical trials using resveratrol treatment in humans had some disappointing outcomes and the difficulties of the clinical application of resveratrol are enormous, such as its poor water solubility, bioavailability, and dosage. Therefore, various strategies are being implemented, which include the development of resveratrol analogues [138] and formulations such as adjuvants, nanoparticles, liposomes, micelles, and phospholipid complexes, to improve its bioavailability. In addition, several other approaches have been employed to enhance its bioavailability, which include altering the route of administering resveratrol and obstructing the metabolic pathways via co-treatment with other agents. In fact, since resveratrol has multiple intracellular targets, additional data is needed to determine the consequences of the interaction or the synergistic effects between other polyphenols and vitamins, amino acids and other micronutrients or ordinarily used drugs. More detailed and well-controlled preclinical and clinical trials are inevitable to evaluate the efficacy of these new formulations as compared with the parental compound. Therefore, further studies in humans are required to improve its bioavailability and to clarify the mechanisms of action of resveratrol in several physiological conditions in order to make this agent a cutting-edge therapeutic strategy for the prevention and treatment of a wide variety of autoimmune and inflammatory chronic diseases.

## Figures and Tables

**Table 1:** Activity and effects of resveratrol in immune cells and in mice models.

Study Type	Subjects	Dose	Effect	Ref.
In vitro	Splenic lymphocytes, CTLs and LAKs	25–50 μM	Suppresses mitogen-, IL-2-, and alloantigen-induced proliferation of splenic lymphocytes; development of antigen-specific CTLs; LAK cells were less sensitive.	[10]
In vitro	T lymphocytes and Macrophages	1–20 µM	Suppresses: T cells proliferation and secretion of IFN-γ and IL-4; B cells proliferation and production of IgG1 and IgG2a isotypes; IL-1, IL-6, TNF-α. Enhances: IL-10; down-regulates the expression of CD28 on CD4^+^ T cells and of CD80 on macrophages.	[13]
In vitro	NK92 cell line	1.5 µM	Enhances perforin expression and cytotoxic activity acting via NKG2D-dependent JNK and ERK-1/2 pathways.	[12]
Ex vivo In vivo	Splenocytes C57BL/6 and BALB/c mice	25–75 µM 4 mg/kg, i.p.	Suppresses the CD4^+^CD25^+^ subsets; downregulated secretion of TGF-β. Enhances IFN-γ expression in CD8^+^ T cells.	[15]
In vitro	RAW 264.7 cell line and BV-2 cell line	50 μM	Suppresses IL-6, M-CSF, MCP-1, MCP-5, CD54, IL-1ra, IL-27, and TNF-α in both cell lines. Inhibits the TLR4/NF-κB/STAT signaling cascade	[20]
In vivo	NOD mice were given	250 mg/kg	Decreases in expression of CCR-6. Inhibits CD11b^+^F4/80^hi^ macrophages. It reduces CCR6^+^ IL-17-producing cells and CD11b^+^F4/80^hi^ in the pancreas. It reduces migration of splenocytes toward media containing CCL20. Prevents type 1 diabetes in NOD mice.	[32]
In vitro	U-937 Jurkat HeLa and H4 cells lines	0.5–25 μM	Suppresses TNF-induced phosphorylation and nuclear translocation of the p65 subunit of NFκ B, and NFκ B-dependent reporter gene transcription. It suppresses TNF-induced NFκ B activation. Blocks NFκ B activation induced by PMA, LPS, H_2_O_2_, and okadaic acid. Suppresses AP-1. Inhibits the TNF-induced activation of MEK and JNK. Abrogates TNF-induced cytotoxicity and caspase activation. Suppresses ROI generation and lipid peroxidation.	[37]
In vitro	Bone-derived cell cultures and MC3T3-E1 cell lines	5 μM	Inhibits RANKL-induced acetylation and nuclear translocation of NFκ B. Induces Sirt1-p300 association in bone-derived and preosteoblastic cells, leading to deacetylation of RANKL-induced NFκ B, inhibition of NFκ B transcriptional activation, and osteoclastogenesis. It activates the bone transcription factors Cbfa-1 and Sirt1 and induces the formation of Sirt1-Cbfa-1 complexes. It regulates the balance between the osteoclastic versus osteoblastic activity. It could exert a therapeutic potential for treating osteoporosis and rheumatoid arthritis-related bone loss.	[38]
In vitro	MH7A cell lines	100 μM	Induces MH7A cell apoptosis by activating caspase-9 and the effector caspase-3, reduces Bcl-XL expression, allowing cytochrome c release from the mitochondria into the cytosol, in a sirtuin 1-dependent manner. It could suppress hyperplasia of synovial cells, a critical factor of rheumatoid arthritis.	[40]
In vitro	RAW264.7 and HEK 293T cell lines	30, 50, 75, 100 μM	Inhibits TRIF signaling in the TLR3 and TLR4 pathway by targeting TANK-binding kinase 1 and RIP1 in TRIF complex. Modulates TLR-derived signaling and inflammatory target gene expression. It could alter susceptibility to microbial infection and chronic inflammatory diseases.	[46]
In vitro	RAW 264.7 cell line	50 μM	Suppresses LPS-induced TRAF6 expression and ubiquitination, attenuates the LPS-induced TLR4–TRAF6, MAPK, and AKT pathways. It could exert anti-inflammatory effects.	[47]
In vitro	Mouse bone-marrow cells J774 cell line	5 μM	Inhibits the accumulation of acetylated α-tubulin and suppressing NLRP3-inflammasome assembly. It prevents the NLRP3-related inflammatory diseases.	[53]
In vitro	AR42J cell line	10–100 μM	It decreases CD14 and IRAK1 expression and increases the p38 MAPK protein phosphorylation. It exerts antioxidant properties either by a Myd88-dependent way not involving IRAK1 or by a TRIF dependent pathway.	[55]
In vitro	RAW 264.7 THP-1 HUVEC cell lines and PBLs	6.25–12.5–25–50 μM 3.13–6.25–12.5–25 μM 10–20–30 μM 6.25–12.5–25 μM	Modulates many mediators of the inflammatory response. Its effects are context-dependent, influencing chemokines and cytokines in opposite ways in different cells.	[58]
In vitro	Macrophages	2.5 μM	Suppresses LPS-induced phosphorylation of FoxO3a. Blocks the LPS-induced PI3K-AKT pathway and affects FoxO3a phosphorylation. Inhibits Nox1 and MCP-1 expression. Could modulate the activations of important macrophage functions associated with atherosclerosis.	[61]
In vitro	TPH1 cell line	25 μM	Promotes apoA-1 and HDL-mediated efflux, downregulates oxLDL uptake, and diminishes foam cell formation. Regulates expression of the cholesterol metabolizing enzyme CYP27A1, and helps cholesterol elimination.	[62]
In vitro	TPH1 cell line	2.5 μM	Inhibits foam cells formation by regulating the expression of the inflammatory cytokine, MCP-1, and by activating the AMPK-Sirt1-PPAR signaling pathway.	[63]
In vitro	Granulocytes Monocytes RAW 264.7 cell line	5–100 μM	Inhibits oxidative burst and CD11b expression in granulocytes and monocytes. Inhibits the production of NO and PGE2, but did not reduce iNOS, TNFα, or IL-1β gene expression in LPS-stimulated RAW 264.7. Induces NRf2 nuclear translocation and reduced miR-146a expression in LPS treated RAW 264.7.	[64]
In vitro	Human rheumatoid arthritis synovial fibroblasts	20 μM	Suppresses the bradykinin-induced COX-2/PGE_2_. Inhibits the phosphorylation and acetylation of p65, c-Jun, and Fos and reduces the binding to the COX-2 promoter, thereby attenuated the COX-2 expression. Could be used for inflammatory arthritis therapy.	[65]
In vivo In vitro	C3H/He mice Splenocytes	1.5 mg/Kg 1.25–2.5–5 μM	Reprograms M-2 phenotype (TAM) countering the immunosuppressive and tumor progressive influences of TAM.	[83]
In vitro	M2 polarization of human monocyte derived macrophages	20 μM	Decreases STAT3. It inhibits F4/80 positive expressing cells and M2 polarization in the tumors.	[86]
In vivo	C3H/He mice	0.5, 1 and 1.5 mg/kg	Reduces Tregs (CD4 ^+^ CD25 ^+^ Foxp3 ^+^ cells) and the production of TGF-β. Increases IFN-γ-expressing CD8 ^+^ T cells. Upregulates IFN-γ production and enhances the cytotoxicity of splenocytes against FM3A tumor cells.	[97]
In vitro In vivo	T cell C57/BL6 and DBA1 mice	0.5 μM or 25 μM 25 mg/kg	Upregulates Sirt1 expression. Decreases c-Jun acetylation and its translocation. Reduces the incidence and severity of collagen-induced arthritis in mice.	[100]
In vivo	Wistar rats	100 mg kg^-1^ ml	Downregulates PKC9 level in T lymphocytes	[101]
In vivo	C57BL/6 mice	HFD supplemented with 0.06% resveratrol	Activates the PI3K and Sirt1 signaling transduction. Activates the Nrf2-regulated adaptive response. Increases the CD3^+^CD4^+^/CD3^+^CD8^+^ subsets percentages and the Tregs. Maintains glucose homeostasis alleviating inflammation.	[104]
In vitro	PBMCs	0.625–2.5–5–10 μM	Modulates the functional activities of both T and NK effector cells, with stimulation at low concentrations and suppression at high concentrations. Affects cytokine-production by activated CD41 and CD81 T cells.	[114]
In vitro	KHYG-1, NKL, NK-92, and NK-YS cell lines	3.125–6.25–12.5– 25–50 μM	Suppresses STAT3 and inhibits JAK2 phosphorylation. Induces downregulation of the anti-apoptotic proteins MCL1 and survivin. Induces apoptotic and antiproliferative activities of L-asparaginase against KHYG-1, NKL and NK-92 cells.	[115]
In vitro	Human NKs Jurkat cell line	0.5−50 μM	At high concentration promotes apoptosis of NK cells and of Jurkat cells. At low concentration increases the NK cells cytotoxicity via up-regulating the expression of NKG2D and IFN-γ.	[116]
In vitro	KG-1a cells PBMCs	25–100 μM	Inhibits KG-1a cell growth but has the least growth-inhibition effect PBMCs. Makes KG-1a cells susceptible to CIKs-mediated cytolysis correlated with an increase in cell-surface expression of NKG2D ligands and DR4, coupled with a downregulation of cell-surface expression of DcR1.	[13]
In vitro	DU145, and PC3 cells	5–30 μM	Induces apoptosis in prostate cancer cells. Downregulates Bcl-2, Bcl-XL, and surviving. Upregulates Bax, Bak, PUMA, Noxa, and Bim, TRAIL-R1/DR4 and TRAIL-R2/DR5 expression. Activates caspase-3 and caspase-9 and induces apoptosis.	[119]
In vitro	cell lines LU120 cell line	25–100 μM	Decreases STAT3 and NF-κB activation. Suppresses expression of cFLIP and Bcl-xL proteins and increases sensitivity to exogenous TRAIL in DR5-positive melanomas. In combination with TRAIL it could have a significant efficacy in the treatment of human melanomas.	[121]
In vitro	HL60 T47D cell line	32 μM	Induces cell death mediated by intracellular caspases Dose-dependent increase in proteolytic cleavage of caspase substrate PARP. Enhances CD95L expression on both HL60 cells T47D breast carcinoma cells.	[123]
In vivo In vitro	BALB/c or C57BL/6 mice tBregs	20 or 50 mg/mouse 12.5 mM	Inhibits lung metastasis in mice. Inactivates Stat3, preventing the generation and function of tBregs, including expression of TGF-β. It reduces antitumor effector immune responses by disabling tBreg-induced conversion of Foxp3^+^ Tregs. Could control cancer escape-promoting tBregs/Tregs without nonspecific inactivation of effector immune cells.	[131]
In vivo	MRL/lpr mice BJAB B cells	20 mg kg^−1^ per day	Increases the expression of FcγRIIB in B cells. Decreases serum autoantibody titers in MRL/lpr mice. The upregulation of FcγRIIB causes an increase of Sirt1 protein and deacetylation of p65 NF-κB. Reduces plasma cells in MRL/lpr mice, leading to improvement of nephritis and prolonged survival.	[132]
In vivo	BALB/c mice	20 mg/kg	Reduces proteinuria, immunoglobulin deposition in kidney, and in serum in pristane-induced lupus mice. Inhibits CD69 and CD71 expression on CD4+ T cells and CD4+ T cell proliferation. Induces CD4+ T cell apoptosis, and decreased CD4 IFNc^+^ Th1 cells and the ratio of Th1/Th2 cells in vitro. Inhibits antibody production and proliferation of B cells in vitro.	[134]

**Abbreviations:** AKT, protein kinase B; AMPK, AMP-activated protein kinase; AP-1, activator protein 1; apoA-1, apolipoprotein (Apo) A-I; Bax, Bcl-2-associated X protein; Bak, Bcl-2 homologous antagonist killer; Bcl-2, B-cell lymphoma; Bcl-xL, B-cell lymphoma-extra-large; Bim, Bcl-2-like 11; Cbfa-1, core-binding factor a1; CCL20, chemokine (C-C motif) ligand 20; CCR 6 chemokine (C-C motif) receptor 6; cFLIP, cellular FLICE-inhibitory protein; CIKs, cytokine-induced killer cells; COX-2, cyclooxygenase-2; CTLs, cytotoxic T lymphocytes; CYP27A1, cytochrome P450 27-hydroxylase; DR, death receptor; DcR1, decoy receptor 1; ERK1/2, extracellular signal–regulated kinases; FcγRIIB, Fc gamma receptor IIb; FoxO3a, forkhead box O3A; Foxp3, forkhead box P3; HDL, high-density lipoprotein cholesterol; HFD, high-fat diet; IFN-γ interferon-gamma; IL, interleukin; iNOS, inducible nitric oxide synthase; IRAK1, interleukin-1 receptor-associate kinase 1; JAK2, janus activated kinase; JNK, c-Jun N-terminal kinase; LAKs, lymphokine activated killer cells; LPS, lipopolysaccharide; MAPK, mitogen-activated protein kinase; M-CSF, macrophage colony stimulating factor; MCP, monocyte chemoattractant protein; MEK, mitogen-activated protein kinase; Myd88, myeloid differentiation factor 88; NK, natural killer; NLRP3, NOD-like receptor family pyrin domain containing 3; NKG2D, natural killer group 2 member D; Nrf2, nuclear factor (erythroid-derived 2)-related factor-2; NF-κB, nuclear factor-kappa B; NOD, nucleotide oligomerization domain; PARP poly (ADP-ribose) polymerase; PBMCs, peripheral blood mononuclear cells; PGE2, prostaglandin E2; PI3K, phosphoinositide 3-kinase; PKCϑ, protein kinase c-delta; PMA, phorbol 12-myristate13-acetate; PPAR, peroxisome proliferator-activated receptors; PUMA, p53 upregulated modulator of apoptosis; RANKL, receptor activator of nuclear factor kB ligand; RIP, receptor interacting protein; ROI, reactive oxygen intermediate, Sirt1, Sirtuin-1; STAT, signal transducer and activator; TAMs, tumor associated macrophages; TANK, TRAF family member-associated NF-κB activator; tBregs, TGFβ-expressing regulatory B cells; Tregs, regulatory T cells; TRAF6, tumor necrosis factor receptor-associated factor 6; TRAIL, tumor necrosis factor-related apoptosis-inducing ligand; TNF-related apoptosis inducing ligand; TRIF, TIR-domain-containing adapter-inducing interferon; TLR, toll-like receptor; TGF-β, transforming growth factor beta; TNF-α, tumor necrosis alpha.
